# Prehospital patients with acute isolated vertigo and imbalance referred for cerebral thrombolysis

**DOI:** 10.3389/fneur.2025.1561202

**Published:** 2025-05-21

**Authors:** S. R. H. De Schryver, M. de Jonge, R. P. van Luxemburg, V. J. Geraedts, E. L. L. M. De Schryver

**Affiliations:** ^1^Department of Neurology, Alrijne Hospital, Leiderdorp, Netherlands; ^2^Department of Neurology, LUMC, Leiden, Netherlands

**Keywords:** isolated vertigo, intravenous thrombolysis, imbalance, stroke, triage

## Abstract

**Introduction:**

Patients with acute vertigo and gait imbalance who are suspected of having a stroke, are increasingly referred to the hospital for intravenous thrombolysis treatment (IVT) with an increasing impact on the healthcare consumption. This study seeks to examine the medical feasibility of considering patients suffering from acute vertigo with imbalance in a prehospital setting without other neurological symptoms as potential candidates for IVT regarding its efficacy and impact on clinical outcomes.

**Methods:**

Patients referred for IVT with isolated vertigo as determined by the ambulance paramedics, were retrospectively enrolled from a single center. Patients were categorized by discharge diagnosis. Baseline characteristics were recorded. The Modified Rankin Score (mRS) was used to assess clinical outcomes.

**Results:**

163 consecutive patients were included. Within this cohort, 5.5% were diagnosed with stroke, and 7.9% with suspected stroke. Among patients with (suspected) stroke, 59.1% received IVT. Among patients with (suspected) vestibular vertigo, 5.1% received IVT. Patients with (suspected) stroke exhibited higher National Institutes of Health Stroke Scale [NIHSS; Mdn 0 (Q1 0; Q3 1) and Mdn 2 (Q1 0; Q3 2.5)]. Additionally, walking ability did not significantly vary between patients with vestibular disease and stroke. The mRS scores after a 3-month period did not show significant differences between the groups.

**Conclusion:**

Accurately differentiating between central and peripheral causes of vertigo in the hyperacute setting is challenging and carries the risk of overreferral and overtreatment. Combined with the lack of evidence that IVT improves clinical outcomes in patients with isolated vertigo and imbalance, and the increasing demand for healthcare, the authors suggest not referring these patients immediately for IVT but to consider adequate training of the paramedics in-field and other routes of medical assessment and treatment.

## Introduction

Even for highly experienced neurologists, it can be challenging to differentiate between central and peripheral causes of vertigo. This difficulty, coupled with the increasing focus on speed in emergency room (ER) settings, has led to a growing practice of referring patients who suffer from acute isolated vertigo with gait imbalance in a prehospital setting to the ER for intravenous thrombolysis treatment (IVT) (1). While clinical findings alone may not be sufficient for a conclusive differentiation, accurately distinguishing between central and peripheral vertigo is crucial for determining the appropriate course of treatment. Even MRI can be false negative in this hyperacute setting (2).

The administration of IVT comes with inherent risks, most notably the causing of intracranial hemorrhage (3), although the risk is significantly lower for stroke mimics and in the vertebrobasilar region (4–7).

There is a scarcity of robust studies examining the specific impact of IVT on posterior stroke, particularly in patients presenting with isolated vertigo or without disabling deficits. In patients with non-disabling stroke, treatment with aspirin resulted in equally favorable functional outcomes as treatment with IVT (8–11) Moreover, ischemia in the cerebellum often has a good prognosis without treatment (6, 8, 12).

Considering the rising demand for healthcare in Western society, driven by an aging population and expanding treatment options, it becomes crucial to utilize care efficiently and prioritize evidence-based medicine. The aim of this study is to validate the question whether referral of patients with isolated vertigo and gait imbalance for IVT is effective care.

## Methods

### Patients and clinical evaluation

Patient data were obtained retrospectively from electronic medical records and based on the natural influx of patients to the ER over a period of one and a half years (from September 1, 2021, to February 28, 2023) at the Alrijne Hospital in the Netherlands.

Inclusion criteria were: (1) referral by paramedics who designated patients as possible eligible for IVT and (2) suffering from acute isolated vertigo with or without walking disorder, with or without nystagmus, without other (focal) neurological symptoms, as determined by the ambulance paramedics.

Baseline characteristics were recorded, as well as stroke risk factors. Patients were defined as non-smokers/ non-alcohol users if they did not consume or had quit for a period longer than 5 years.

Based on the diagnose made by the treating neurologist at discharge from the hospital, patients were classified into one of the following categories: (1) confirmed vestibular vertigo, (2) suspected vestibular vertigo, (3) suspected stroke, (4) confirmed stroke and (5) non-vestibular non-stroke causes of vertigo. The presence of a stroke was established by confirming the existence of a visible lesion on CT-, CT-perfusion, CT-angiography or MRI-scan that was consistent with a recent stroke, or by identifying other focal neurological symptoms that were consistent with a stroke. Patients were categorized as suspected stroke cases if they exhibited clinical symptoms consistent with stroke, despite the absence of visible abnormalities on imaging.

The confirmation of a vestibular cause was based on several factors, including the presence of third-degree nystagmus in combination with positive results from the HINTS examination, vestibular provocation testing, and a thorough assessment of the patient’s recent clinical history. Patients were categorized into the suspected vestibular group if they displayed no evidence of stroke but did not exhibit a fully positive HINTS examination.

Special attention was given to the presence of focal neurological symptoms such as ataxia and a detailed description of the patient’s gait. The degree of walking ability was prospectively thoroughly documented and classified in accordance with local protocol. Gait was categorized into four distinct categories: (1) ability to walk independently without assistance, (2) ability to walk with minimal support, (3) ability to walk with the assistance of two individuals, and (4) inability to walk, but able to sit without assistance.

The patient’s outcome was assessed with the modified Rankin Score (mRS) at the time of initial presentation and during the follow-up period after 3 months during the regular check-up appointment for stroke patients. The National Institutes of Health Stroke Scale (NIHSS) was recorded at the time of presentation, with only new complaints being counted in the final score. When evaluating the mRS, both pre-existing complaints and new complaints were taken into consideration.

This study was approved by the Board of Directors of the Alrijne Hospital. As the research involves a retrospective analysis of de-identified data collected from September 1, 2021, to February 28, 2023, the requirement for informed consent was waived in accordance with the Medical Research Involving Human Subjects Act (WMO). All data analyzed were anonymized to protect patient privacy.

### Statistical analysis

All statistical analyses were performed using IBM SPSS version 28. Descriptive statistics such as median and interquartile range (IQR), were analyzed using a Kruskal-Wallis test on ranks. A non-parametric test was chosen, given the non-normal distribution of the data.

Nominal data were analyzed using Chi square Fisher’s exact tests. Pairwise comparisons were analyzed using Mann–Whitney U test and adjusted for significance with the Bonferroni correction.

## Results

### Patient characteristics

Out of a total of 163 patients, 9 individuals were diagnosed with stroke, 13 patients had suspected stroke, 88 were diagnosed with vestibular disease, 30 patients had suspected vestibular vertigo. Of the 9 patients with stroke, only 1 patient had isolated vertigo with imbalance. Eight patients had focal neurological symptoms such as diplopia, hemiataxia and dysarthria. Twenty-four patients were included in the category of non-vestibular non-stroke causes of vertigo, which consisted of varying conditions such as having suffered an epileptic seizure or reflex syncope, but also symptoms related to orthostatic hypotension or general feeling of malaise.

No significant differences in stroke risk factors were observed between the stroke and vestibular groups (Table 1).

**Table 1:** Baseline characteristics.

Sociodemographic/clinical characteristics	Vestibular	Suspected vestibular	Suspected stroke	Stroke	Non-vestibular / non-stroke	*p*-value
Total (N)	88 (53.7%)	30 (18.3%)	13 (7.9%)	9 (5.5%)	24 (14.6%)	
Age (Mdn)	Mdn 68.5 (Q1 60; Q3 77)	Mdn 73.0 (Q1 61; Q3 85)	Mdn 80.0 (Q1 75; Q3 82.5)	Mdn 77.0 (Q1 57; Q3 83)	Mdn 70 (Q1 53.5; Q3 76.50)	0.044^†^^,^^a^
Sex (M)	43 (48.9%)	12 (40.0%)	5 (38.5%)	6 (66.7%)	8 (33.3%)	0.406^‡^
Hypertension	31 (36.0%)	14 (46.7%)	7 (53.7%)	4 (50.0%)	15 (62.5%)	0.180^‡^
Hypercholesterol emia	24 (27.9%)	8 (26.7%)	5 (38.5%)	4 (50.0%)	3 (12.5%)	0.222^‡^
DM	10 (11.8%)	3 (10.0%)	2 (15.4%)	1 (12.5%)	1 (4.2%)	0.764^‡^
Smoking	9 (27.3%)	0 (0%)	1 (33.3%)	1 (25.0%)	5 (31.3%)	0.072^‡^
Alcohol	17 (56.7%)	6 (37.5%)	3 (100%)	3 (60.0%)	11 (78.6%)	0.128^‡^
Vitamin K antagonist	6 (9.2%)	3 (21.4%)	0 (0%)	0 (0%)	0 (0%)	0.269^‡^
DOAC	5 (7.8%)	2 (15.4%)	1 (16.7%)	0 (0%)	1 (5.3%)	0.570^‡^
Antiplatelet agents	17 (22.4%)	14 (56.0%)	6 (54.5%)	1 (12.5%)	4 (18.2%)	0.004^‡^^,^^b^
History of stroke	14 (16.7%)	14 (50.0%)	4 (30.8%)	0 (0.0%)	5 (22.7%)	0.005^‡^^,^^c^

Mdn, median; IQR, interquartile range; DM, diabetes mellitus; DOAC, direct oral anticoagulant.

† Kruskal Wallis H.

‡ Chi2 exact. Group 1 = vestibular, 2 = suspected vestibular, 3 = suspected stroke, 4 = stroke, 5 = non-vestibular, non-stroke.

a No significant between-group differences.

b Group 1 and 2 differed significantly (*p* = 0.026).

c Group 1 and 2 differed significantly (*p* <0.001).

### Neurological examination

The patients with suspected stroke and confirmed stroke had a significantly higher NIHSS [Mdn 0 (Q1 0; Q3 1) and Mdn 2 (Q1 0; Q3 2.5)], due to concomitant neurological symptoms such as ataxia, diplopia and/or dysarthria which were observed in 8 stroke patients.

Neither the ability to walk without help nor the presence of nystagmus differed significantly between vestibular disease and stroke as the cause of vertigo (Table 2).

**Table 2:** Outcomes.

Neurological examination	Vestibular	Suspected vestibular	Suspected stroke	Stroke	Non-vestibular/ non-stroke	*p*-value
Total	88 (54%)	30 (18%)	13 (8%)	9 (5%)	24 (15%)	
NIHSS ER	Mdn 0 (Q1 0; Q3 0)	Mdn 0 (Q1 0; Q2)	Mdn 0 (Q1 0; Q3 1)	Mdn 2 (Q1 0; Q3 2.5)	Mdn 0 (Q1 0; Q3 0)	<0.001^†^^,^^a^
mRS ER	Mdn 1 (Q1 1; Q3 3)	Mdn 1 (Q1; Q3 1.25)	Mdn 2 (Q1 1; Q3 4)	Mdn 2.5 (Q1 1; Q3 4)	Mdn 1 (Q1 1; Q3 1)	0.024^†^^,^^b^
Gait 1	57 (64.8%)	20 (66.7%)	9 (69.2%)	2 (22.2%)	19 (79.2%)	0.058^‡^
Gait 2	14 (15.9%)	5 (16.7%)	0 (0%)	1 (11.1%)	3 (12.5%)	0.674^‡^
Gait 3	7 (8.0%)	2 (6.7%)	2 (15.4%)	3 (33.3%)	0 (0.0%)	0.042^‡^^,^^b^
Gait 4	3 (3.4%)	1 (3.3%)	2 (15.4%)	3 (33.3%)	0 (0.0%)	0.007^‡^^,^^b^
Able to walk independently	24 (29.6%)	8 (28.6%)	4 (30.9%)	7 (77.8%)	3 (13.6%)	0.017^‡^^,^^c^
Ataxia	0 (0.0%)	1 (3.3%)^§^	1 (7.7%)^∥^	6 (66.7%)	0 (0.0%)	0.000^‡^^,^^f^
Nystagmus	57 (64.8%)	13 (43.3%)	4 (30.8%)	7 (77.8%)	3 (12.5%)	0.000^‡^^,^^d^
3rd degree nystagmus	33 (37.5%)	2 (6.7%)	2 (15.4%)	0 (0.0%)	0 (0.0%)	0.000^‡^^,^^e^
Positive HIT	31 (60.8%)	7 (43.8%)	1 (25.0%)	0 (0.0%)	0 (0.0%)	0.002^‡^^,^^g^
Missing	37	14	9	5	17	
Treatment and follow up						
IVT	4 (4.5%)	2 (6.7%)	5 (38.5%)	8 (88.9%)	0 (0%)	0.000^‡^^,^^h^
mRS during follow-up	–	*n* = 5 Mdn 1 (IQR 0.5)	*n* = 11 Mdn 1 (IQR 0.0)	*n* = 8 Mdn 1 (IQR 1.5)	–	0.704^‡^

Mdn = median, IQR = interquartile range. NIHSS = National Institutes of Health Stroke Scale, ER = emergency room, mRS = modified Rankin Scale, HIT = head impulse test, CT = computerized tomography, IVT = intravenous thrombolysis. Gait: (1) ability to walk independently without assistance, (2) ability to walk with minimal support, (3) ability to walk with the assistance of two individuals, and (4) inability to walk.

† Kruskal Wallis H.

‡ Chi2 exact.

§ pre-existent.

∥ dubious. Group 1 = vestibular, 2 = suspected vestibular, 3 = suspected stroke, 4 = stroke, 5 = non-vestibular, non-stroke.

a significant differences between group 1–4 (*p* = 0.000); 2–4 (*p* = 0.000); 3–4 (*p* = 0.003); 5–4 (*p* = 0.000).

b no significant between-group differences.

c significant differences between group 1–5 (*p* = 0013).

d significant differences between group 1–5 (*p* <0.001); 4–5 (*p* <0.001).

e significant differences between group 1–2 (*p* = 0.010); 1–5 (*p* <0.001).

f significant differences between group 1–4 (*p* <0.001); 2–4 (*p* <0.001); 4–5 (*p* <0.001).

g significant differences between group 1–5 (*p* = 0.030).

h significant differences between group 1–3 (*p* = 0.015); 1–4 (*p* <0.001); 2–4 (*p* <0.001); 3–5 (*p* = 0.030); 4–5 (*p* <0.001).

Eight patients had a positive peripheral HINTS exam, characterized by a positive Head Impulse Test (HIT) and unidirectional or no nystagmus without skew deviation. All these patients were in the suspected vertigo group. It is likely that the HINTS exam was performed more frequently than documented. In the entire cohort, there were no patients with a positive skew test, indicating the absence of patients with a positive central HINTS.

### Diagnosis and treatment

Among the 163 patients who presented to the emergency room with acute vertigo, 19 received IVT. Out of these 19 patients, 6 were diagnosed with (probable) vestibular disease at discharge. None of them had adverse effects.

### Imaging

In the vestibular group, of the 75 scans performed, 1 showed bilateral subdural bleeding. This was considered an incidental finding in addition to the diagnosis of peripheral vestibulopathy.

In the stroke group, all patients had brain-imaging, on which no hemorrhage was visible. Two in seven stroke patients had relevant abnormal findings on their CT angiograms: a right vertebral artery dissection and a partial right basilar artery occlusion with compatible ischemia on CT-perfusion. Two stroke patients had abnormal findings on MRIs: a small lacunar ischemia and a possible small infarction of the right cerebellum.

### Follow-up

Of the 9 patients who were classified as certainly having suffered from stroke, 8 received IVT. After 3-month follow-up, the 9 patients had a median mRS of 1 (Q1 0.25; Q3 1.75), meaning that most patients had no or little remaining complaints (with walking or dizziness) without significant disability. The patient with stroke that did not receive IVT, had an mRS of 1 during follow-up. One patient in this group had an mRS of 4 and suffered from dysarthria and hemi-ataxia on arrival at the ER. The mRS did not differ significantly between the suspected vestibular and (suspected) stroke groups.

## Discussion

The prevalence of stroke in patients presenting primarily with vertigo varies enormously across studies, ranging from 0.7 to 60% (13, 14). The highest percentage was observed in a study performed in a tertiary stroke center which had earlier reported a prevalence of 5%. According to the authors, the percentage of stroke prevalence increased due to a radical reorganization in stroke care in their region (13, 14). The majority of studies, however, report a prevalence of around 5% (1, 15). In this study, the prevalence of patients with isolated vertigo and (suspected) stroke was 12%.

Similarly, the percentage of patients receiving IVT after primarily presenting with vertigo also varies among studies, ranging from <5% up to 53% (16, 17). Furthermore, it is likely that IVT is frequently administered to vertigo patients with stroke mimics (17, 18). Several studies have indicated that IVT does not significantly impact short-term prognosis in patients with mild ischemic stroke presenting with vestibular symptoms (6, 8–12, 16, 17).

Even for IAT, there is no clear evidence in posterior large vessel occlusion which indicates a difference between posterior circulation and the anterior circulation in terms of treatment prognosis (19, 20).

### Limitations

This study focused on patients with acute isolated vertigo presenting for IVT to the ER. This specific population was selected due to the resource-intensive and costly nature of such healthcare interventions, taking into account the limitation of a good neurological evaluation in a pre-hospital setting. Among the 163 patients included, neurological examinations at the emergency room identified additional focal neurological symptoms other than dizziness and gait imbalance in 8 individuals (5%). These patients should have been excluded from the study. Due to the nature of this study, it cannot be ruled out that these accompanying symptoms were in fact observed, but not properly documented in the admission files of the ambulance or ER personnel during acute care. Another way to frame the analysis done in this paper, is to categorize the included patients as having isolated vertigo and possibly other non-disabling focal neurologic deficits. Symptoms that are extremely subtle and likely to be overlooked are often (but not always) not very disabling and therefore may make thrombolysis less justifiable. This is aligned with how many stroke neurologists approach the thrombolysis decision.

Assessing gait disturbances in an acute situation can be challenging due to factors such as nausea and dizziness. Although we had an insufficient number of patients to draw firm conclusions regarding severity of imbalance, our study found that severity was not discriminative between vestibular disease and stroke in a hyperacute setting and definitively not pathognomonic for stroke. Previous studies found that a severe imbalance could be correlated with stroke. These studies, however, were either not performed in a hyperacute setting or did not investigate patients with isolated vertigo and/or imbalance (21, 22).

The management of mild strokes with low NIHSS scores and non-disabling symptoms varies significantly in clinical practice. Isolated dizziness without significant gait impairment is typically not considered disabling, and thrombolysis is generally not recommended for NIHSS 0 patients. However, in some cases, severe imbalance that prevents standing or ambulation without significant assistance (gait 3 or 4) is considered disabling by some vascular neurologists, who may still consider thrombolysis in these cases. This variability in clinical practice underscores the importance of further investigation into the role of gait assessment in decision-making.

As this study is a single-center retrospective analysis, we refrain from drawing conclusions regarding the effects of IVT on vertigo. Nonetheless, we intend to highlight that previous research has not provided evidence supporting thrombolysis for vertigo.

Furthermore, in patients with minor non-disabling acute ischemic stroke, treatment with aspirin resulted in equally favorable functional outcomes as treatment with IVT (9–11, 16, 23).

Considering the inherent intensity and costs of care associated with IVT, along with the lack of evidence and risk of misdiagnosis, the authors advocate for the development of local protocols to guide the decision-making process regarding the referral of patients with isolated vertigo for IVT treatment.

A potential limitation of this study is its selection bias of only including patients found eligible for IVT in a prehospital setting by paramedics in-field. However, focusing on these IVT-eligible patients is intentional as it aligns with our objective of examining this specific group to potentially reduce costs and enhance efficacy of available care. Additionally, not all patients received an MRI scan after 24 h, leading to a group comprising both suspected vestibular and suspected stroke patients. For these individuals, the MRI outcomes would not have influenced clinical treatment decisions after the emergency treatment.

Another limitation of this study is the exclusion of initial nystagmus and HINTS examination. The challenging nature of these assessments often causes them to be either not conducted or properly executed by paramedics in a hyperacute setting. In the Netherlands, paramedics and general practitioners currently lack the proficiency of neurologists in performing these examinations. In light of this, the question remains whether it is feasible to develop an effective protocol with an initial nystagmus and HINTS examination by paramedics in-field or to develop other routes for assessment and treatment of these patients instead of immediate referral for IVT.

## Conclusion

It is important to consider stroke as a possible cause of acute vertigo. Acute isolated vertigo, however, is difficult to diagnose accurately in a hyperacute setting, which increases the risk of overtreatment. Moreover, thrombolysis for stroke patients with isolated vertigo with gait imbalance has so far not been proven to be an effective treatment, and further trials are needed. This, combined with the need for efficient use of healthcare resources due to increased consumption, leads the authors to recommend that the assessment and treatment routes of patients with acute isolated vertigo should be carefully developed and thoroughly assessed.

## Data Availability

The dataset supporting the conclusions of this article can be obtained upon reasonable request via corresponding author.
